# Activity of cyclosporins as resistance modifiers in primary cultures of human haematological and solid tumours.

**DOI:** 10.1038/bjc.1994.242

**Published:** 1994-07

**Authors:** H. Fridborg, B. Jonsson, P. Nygren, K. Csoka, K. Nilsson, G. Oberg, J. Kristensen, J. Bergh, B. Tholander, L. Olsen

**Affiliations:** Division of Clinical Pharmacology, University Hospital, Uppsala University, Sweden.

## Abstract

The semiautomated fluorimetric microculture cytotoxicity assay (FMCA) was used for evaluation of the ability of cyclosporin A (CsA) and its novel non-immunosuppressive derivative SDZ PSC 833 (PSC) to modify the response to doxorubicin or vincristine in vitro in different haematological and solid human tumour types. Primary cultures of 322 tumour samples were analysed. Both cyclosporins showed resistance-modifying activity in all haematological tumours tested, and in solid tumours activity was observed in ovarian carcinoma and childhood tumours. Little or no effect was found in the remaining tumour types, including breast, renal and adrenal cortical carcinomas and adult sarcomas. In most of the responsive cases the interaction between the modifier and the cytotoxic drug was synergistic. There was a tendency to higher activity in samples from previously treated patients, and an inverse relationship between degree of cytotoxic drug resistance and resistance-modifying activity was noted. No difference in potency between CsA and PSC could be discerned. The results indicate differential in vitro resistance-modifying activity of the cyclosporins depending on tumour type. The results also suggest that treatment with resistance modifiers should be considered also for primary therapy of drug-sensitive tumours. Drug resistance assays such as the FMCA may become useful in preclinical evaluation of resistance modifiers.


					
Br. J. Cancer (1994). 70, 11-17                                                                      C  Macmillan Press Ltd., 1994

Activity of cyclosporins as resistance modifiers in primary cultures of
human haematological and solid tumours

H. Fridborg', B. Jonsson', P. Nygren2, K. Csoka', K. Nilsson3, G. Oberg4,

J. Kristensen4, J. Bergh2, B. Tholander5, L. Olsen6, A. Jakobson7 & R. Larsson'

'Division of Clinical Pharmacology and Departments of 2Oncology, 'Pathology, 'Medicine, 'Gynecological Oncology, 6Paediatric
Surgery and 'Paediatrics, University Hospital, Uppsala University, 5-751 85 Uppsala, Sweden.

S   ary   The semiautomated fluorimetric microculture cytotoxicity assay (FMCA) was used for evaluation
of the ability of cyclosporin A (CsA) and its novel non-immunosuppressive derivative SDZ PSC 833 (PSC) to
modify the response to doxorubicin or vincristine in vitro in different haematological and solid human tumour
types. Primary cultures of 322 tumour samples were analysed. Both cyclosporins showed resistance-modifying
activity in all haematological tumours tested, and in solid tumours activity was observed in ovarian carcinoma
and childhood tumours. Little or no effect was found in the remaining tumour types, including breast, renal
and adrenal cortical carcinomas and adult sarcomas. In most of the responsive cases the interaction between
the modifier and the cytotoxic drug was synergistic. There was a tendency to higher activity in samples from
previously treated patients, and an inverse relationship between degree of cytotoxic drug resistance and
resistance-modifying activity was noted. No difference in potency between CsA and PSC could be discerned.
The results indicate differential in vitro resistance-modifying activity of the cyclosporins depending on tumour
type. The results also suggest that treatment with resistance modifiers should be considered also for primary
therapy of drug-sensitive tumours. Drug resistance assays such as the FMCA may become useful in preclinical
evaluation of resistance modifiers.

Multidrug resistance (MDR) defines a cellular phenotype in
which the development of resistance in vitro to one cytotoxic
drug confers cross-resistance to other structurally and fumc-
tionally dissimilar drugs (Borst, 1991; van Kalken et al.,
1991). The resistance pattern of the classical MDR pheno-
type often includes the anthracyclines and the vinca alklaloids
and is often associated with increased expression of the
membrane-bound 170 kDa P-glycoprotein (Pgp), coded for
by the MDR] gene. Pgp is believed to function as an ATP-
dependent efflux protein that actively extrudes the drugs from
the tumour cell (Borst, 1991; van Kalken et al., 1991). For at
least some tumour types, Pgp has been implicated in clinical
drug resistance and prognosis (van Kalken et al., 1991).

In vitro, MDR may be circumvented by a variety of non-
cytotoxic agents, including calcium channel blockers, cyclo-
sporins, phenothiazines, neuroleptics and cephalosporins
(Ford & Hait, 1990). The prospect of reversing MDR in the
clinical setting has therefore attracted considerable interest,
and some clinical pilot studies using verapamil have been
promising (Dalton et al., 1989; Miller et al., 1991). Although
verapamil is probably the most well-studied resistance modi-
fier (RM), cardiovascular side-effects preclude its clinical use
at in vitro active concentrations (Pennock et al., 1991). Cyclo-
sporin A (CsA), on the other hand, has been shown to
reverse resistance in vitro at concentrations readily achievable
in vivo without serious toxic effects (Twentyman, 1988;
Sonneveld et al., 1992). Furthermore, a non-immunosuppres-
sive cyclosporin analogue denoted SDZ PSC 833 (PSC) was
recently found to be 10-fold more potent than CsA as an
RM in MDR cell lines (Gaveriaux et al., 1991). The cyclo-
sporins may thus be well suited for testing the concept of
resistance modification in the clinic. Indeed, some pre-
liminary studies have indicated clinical resistance-modifying
activity of CsA in myeloma (Sonneveld et al., 1992) and
acute myelocytic leukaemia (AML; List et al., 1993), and
phase II trials of both CsA and PSC are ongoing.

Recently some preliminary evidence was published suggest-
ing that diagnosis-specific activity could be accurately
detected in accordance with clinical experience by non-
clonogenic drug resistance assays based on measurement of
cell kill in the whole tumour cell population (Weisenthal,

1991; Weisenthal et al., 1991). Corroborating these findings,
we have recently found that the FMCA retrospectively can
detect diagnosis-specific drug activity for a series of standard
drugs (Nygren et al., 1993) and prospectively for 2-
chlorodeoxyadenosine (CdA) (Larsson et al., 1994) and taxol
(submitted for publication). The present study was therefore
undertaken to investigate the relative tumour type-specific
resistance-modifying activity of CsA and PSC in combination
with doxorubicin (Dox) or vincristine (Vcr) in vitro, using a
broad spectrum of tumour diagnoses.

Materias ad methods

Twnour samples and cell preparation

A total of 322 tumour samples from patients with AML,
acute lymphocytic leukaemia (ALL), chronic lymphocytic
leukaemia (CLL), chronic myelocytic leukaemia (CML),

myeloma, non-Hodgkin's lymphoma (NHL) or different

types of solid tumours were included in the study. The
samples were obtained from routine surgery, diagnostic
biopsy or bone marrow/peripheral blood sampling. In some
cases tumour sampling was performed for in vitro drug sensi-
tivity testing only, which was approved of by the research
ethical committee at the Uppsala University Hospital. The
number and characteristics of the samples are detailed in
Table I. Tumour tissue from solid tumours was minced into
1 mm3 pieces and tumour cells were then isolated by col-
lagenase dispersion and Percoll (Kabi-Pharmacia, Uppsala,
Sweden) density gradient centrifugation as described
previously (Nygren & Larsson, 1991). Leukaemic cells were
obtained from bone marrow or peripheral blood by
1.077 g ml-' Ficoll-Paque (Kabi-Pharmacia) density gradient
centrifugation (Larsson et al., 1992a). Viability was deter-
mined by trypan blue exclusion test and the proportion of
tumour cells was judged by inspection of May-Griinwald-
Giemsa-stained cytocentrifuge preparations by a trained
cytopathologist.

Culture medium RPMI-1640 (Northumbria Biologicals,
Cramlington, UK) supplemented with 10% heat-inactivated
fetal calf serum (FCS; Northumbria), 2 mM glutamine,
50Lg ml-' streptomycin and 60 ig ml ' penicillin (Northum-
bria) was used throughout. Cells were cryopreserved in cul-
ture medium containing 10% dimethyl sulphoxide (DMSO;

Correspondence: R. Larsson.

Received 27 October 1993; and in revised form I February 1994.

Br. J. Cancer (1994), 70, 11-17

(C) Macmillan Press Ltd., 1994

12     H. FRIDBORG et al.

Sigma, St Louis, MO, USA) and 50% FCS by initial freezing
for 24 h at - 70'C followed by storage in liquid nitrogen.
Both fresh and cryopreserved samples were used in this
study.

Reagents and drugs

Fluorescein diacetate (FDA; Sigma) was dissolved in DMSO
and kept frozen (-20'C) as a stock solution (lOmgml-')
protected from light. CsA and PSC, provided by P. Anderson
(Sandoz, Basle, Switzerland), were dissolved in absolute
ethanol-phosphate-buffered saline (PBS) and further diluted
in PBS. The final concentration of ethanol never exceeded
0.1%, which had no effect on cell survival. Dox was obtained
from Farmitalia Carlo Erba (Milan, Italy) and Vcr from
Lilly (Giessen, Germany). CsA and PSC were generally tested
at two concentrations, 1 and 3 ;Lg ml', whereas Dox and
Vcr were tested at 0.5 and 0.1 Lg ml-' respectively, cut-off
concentrations empirically derived (EDCCs) as previously
described (Larsson et al., 1992a). Experimental plates were
prepared with 20 #1 per well of drug solution at ten times the
desired final concentration with a programmable pipetting
robot (Pro/Pette; Pernin Elmer, Norwalk, CT, USA). The
plates were stored frozen at -70'C until further use. Drug
stability during storage conditions was estimated by repeated
testing of sensitive cell lines (to be published). CsA and PSC
could be stored in this way for at least 2 months. The
experiments were performed with continuous drug exposure.

Equipment

The 96-well scanning fluorometer (Fluoroscan 2; Flow,
Herts, UK) is equipped with a xenon lamp and broadband
interference filters exciting fluorescence at 485 nm for FDA.
The emitted light from a vertical light path of each well is
sequentially read at 538 nm. One plate is read in approxi-
mately 1 min. In most experiments, cells, medium and drugs
were added to the wells by the pipetting robot, Pro/Pette.
Addition of buffer and fluorochrome was performed with an
automatised 96-well dispenser (Multidrop, Flow).

Cytotoxicity assay procedure

The principal steps of the FMCA procedure have been de-
scribed previously (Larsson et al., 1992a). The assay is based
on measurement of fluorescence generated from hydrolysis of
FDA to fluorescein by cells with intact plasma membranes.
On day 1 180IL per well of the tumour cell preparation
(0.5-5 x 1IO cells per ml of culture medium) was seeded into
the wells of V-shaped 96-well microtitre plates (Nunc, Ros-
kilde, Denmark) prepared as described above. Six blank wells
received only culture medium and six wells with cells, but
without drugs, served as control. The plates were then
incubated at 37C in a humidified atmosphere containing
95% air and 5% carbon dioxide. At the end of the incuba-
tion period (72 h), the medium was removed and the cells
washed with PBS. PBS at I00 Il per well containing FDA
(10 L.g ml-') was then added columnwise to control, experi-
mental and blank wells and the plates were incubated for 1 h
before reading the fluorescence in the Fluoroscan 2. The
fluorimeter was blanked against wells containing PBS includ-
ing the dye but without cells.

Quality control

Quality criteria for a successful assay included > 70%
tumour cells in the cell preparation prior to incubations a

fluorescence signal in control cultures of more than five times
mean blank values and a coefficient of variation (CV) in
control cultures of <30%. The overall success rate of the
assay was approximately 85% for haematological tumours
and 60% for solid tumours with too low a proportion of
tumour cells in the cell preparation being the most common
cause of assay failure. Other causes were low yield of cells

Q)

0

1-
0

;)

._

0

go
f.1
l-

,o
0

6)
u

._

0

-o

la

6)

0.

E

0e

Z:5
an
.0
U

0

an
6)

-
0
I

U
:n
u

:ZI
cc

C14 W0 0   "1-

000

o;Q - r'

ro -
as0       0l

0

0-

0;.E

>.
X

IN VITRO RESISTANCE-MODIFYING ACTIVITY OF CYCLOSPORINS

after separation or a low fluorescence signal in controls. Only
successfully analysed samples are reported here.

Quantification of FMCA results

The results obtained by the indicator FDA are presented as
survival index (SI), defined as the fluorescence of experiment-
al cultures expressed as a percentage of that of control
cultures with blank values subtracted. Since for each sample
the single drugs and the combinations were tested on the
same microtitre plate, and since the CV in test wells is
independent of the SI level, the mean intra-assay CV (CV in
controls within a plate) was used to define response to RMs.
The RM response rate was thus defined as the proportion of
samples showing a >25% (2-3 times mean intra-assay CV
in controls) decrease in SI for Dox or Vcr after addition of
RM at the EDCC in a particular sample, and was divided
into two groups, a 25-50% (+) and a >50% (4-5 times
mean control CV) decrease (+ +). Drug interaction analysis
was performed using the multiplicative concept (Valeriote &
Lin, 1975), in which the effect of an additive combination is
expected to be equal to the product of the effect of its
constituents, whereas synergy is observed when the effect is
greater than this product. Thus, for a two-drug additive
combination composed of single agents with SI values of
50% and 40%, the combination would be expected to result
in an SI value of 20% (0.5 x 0.4). The ratio of observed SI
values and those expected according to an additive interac-
tion model was then calculated and observed/expected ratios
<0.8 were defined as synergistic interactions (Ying Tan et
al., 1988). In some analyses the cytotoxicity data for Dox
were divided into three groups based on the median SI value
and standard deviation (s.d.) (Weisenthal et al., 1991). Values
less than the median were termed low drug resistance (LDR),
values greater than the median but less than the median + 1
s.d. intermediate drug resistance (IDR) and values greater
than the median + 1 s.d. were termed extreme drug resistance
(EDR). Previous studies relating in vitro assay results to
clinical outcome have shown that when the individual SIs for
a particular cytotoxic drug in a large number of samples are
divided into three statistically defined categories as above,
different clinical response probabilities for these groups are
observed. The LDR category has a probability of clinical
response to the particular drug higher than expected, IDR a
lower and EDR an extremely low probability of response
(Bosanquet, 1991; Larsson & Nygren, 1993).

Statistical analysis

SI values were compared using the Student's t-test. Correla-
tions were performed using Pearson's correlation coefficient
and differences in proportions were analysed by the approxi-
mate normal test for comparison of proportions (Colton,
1974). The level of significance was set to P<0.05.

Results

Effect of single drugs

The effects of Dox, Vcr, CsA and PSC on SI for haemato-
logical and solid tumours are shown in Figure 1. For Dox
and Vcr mean values ? standard error of the mean (s.e.m.)
for all samples were 53 ? 1.7 and 70 ? 1.5 respectively. The
group of haematological samples showed lower mean SI
values than the solid tumour group in response to Dox
(42 ? 1.7 and 83 ? 2.8 respectively) and Vcr (66? 1.7 and
87 ? 2.5) and these differences were highly significant
(P<0.001). At 1 ggml-' CsA or PSC little or no effect was
discernible for any of the tumour groups. At 3 pg ml- , CsA
showed increased activity in NHL/CLL and AML/CML/
myeloma with mean SI values of 79 ? 8.3 and 82 ? 3.4
respectively. At this concentration 22% of the NHL/CLL
samples had an SI <50%, which should be compared with
the 0% and 10% for the solid and AML groups respectively

Dox 0.5

Vcr 0.

CsA 1.0
CyA 3.0

K"lZzzzozztzzzottttttE

PSC 1.0
PSC 3.0

0      20     40     60      80

Survival index (%)

I

100

120

Fgwe 1 Effect of CsA, PSC, Dox and Vcr in samples from
patients with ALL (_; n = 15-76), AML/CML/myeloma
(=; n=50-111), NHL/CLL ( ;       n=18-62) and solid
tumours ( E; n = 48-95). The results are presented as mean
values ? s.e.m.

(not shown). PSC at 3 tLg ml-' had effects similar to CsA in
the AML and ALL groups, but slightly weaker effect in
NHL/CLL.

Activity of CsA and PSC as RMs

Haematological tumours showed high and concentration-
dependent response rates to addition of RM to both Dox
and Vcr. The concentration dependence of CsA and PSC was
especially notable for NHL with 86- 100% response at the
highest concentration. Solid tumours were generally less re-
sponsive, with the exception of paediatric solid tumours and
to a lesser extent also ovarian carcinoma, in which response
rates of 19% or more were observed at the lowest concentra-
tion of CsA or PSC in combination with Dox. In contrast,
samples of renal carcinoma and adrenocortical carcinoma
showed no responses at any concentration or combination.
In the solid tumour groups, CsA and PSC appeared less
effective in combination with Vcr than with Dox (Tables II
and III). The differences between the group of haemato-
logical tumours and the solid tumour group with respect to
in vitro response rates to addition of RM were statistically
significant for all combinations (P<0.001-0.05). When all
diagnoses were ranked for the resistance-modifying activity
of CsA and PSC, an overall correspondence between CsA
and PSC activity was observed. The top six ranked diagnoses
in repsonse to CsA thus corresponded to those most respon-
sive to PSC in all cases for Dox, and in five out of six cases
for Vcr. Renal and adrenocortical carcinoma showed the
lowest rank- order for both CsA and PSC in combination
with either Dox or Vcr. The most notable difference between
CsA and PSC was the higher rank order for paediatnc solid
tumours for the resistance-modifying activity of PSC com-
pared with that of CsA.

Th'pe of interaction

There was a good correspondence between the RM response
rates and the proportion of samples showing a synergistic
interaction between the RM and Dox or Vcr (Table IV). The
percentage decrease in cytotoxic drug-induced SI after addi-
tion of RM and the observed/expected ratios, calculated on
the basis of the multiplicative concept for drug interactions,
showed a strong and significant negative correlation. The
highest correlation coefficients were observed for PSC at both
concentrations and for CsA at the lower concentration.
Slightly weaker correlations were noted for CsA at 3 iLg ml-'
(Table IV).

Relationship to previous treatment

In Figure 2 tumours were grouped according to treatment
status and analysed with respect to response rates to the

I                                          --r"

. 1

1                                        --__1-4

I

L

.-j

13

ir-

I

I                                                         ll?                                                                              iw-

112
- I                                I                               I                               I

14    H. FRIDBORG et al.

0 O___

t...0 _1

-~          0 >OO-

I-

_O          0

0

rli

0

on

a-

-

CD

on *O 4  a, 0 o e o   0D  0  0

" C4 "   -      r4           C 4

I--

Qo   -  -_     W  0   .--  0

IRT ro- (7 "   " CD   (O

n-- all -      r i o " -  o
V 000   - CD 0   CD - CD-

-  (. - 0 0 - 0 0 -  0

F-
F-

CIA

-  -   ~ ~ ~ ~ ~ ~ ~ ~ a

% oo  0O   - -4   CO  0  (.4
0 1   o 0  -   I-T- No  -

t ~ ~ ~~~I  CD CD--   -  lq

in CD os  0 a o   o
--o-fo~~C soo Ns v

~00

-t-?- 0     0    0-
- -t -..0 O .E- -a - F- - _

-0 t .400-00- 0

t    0u   aj 0000-^ ^
-  tI- -t%  -  -  -O O- -

- 0- r  -)  F  1-  (.4  (.  0 0 -E_

a-
(.4

(.4

(-.
C1

S

C4? f4  VV   e4 - r4

-? - -- o0 -- 0 -~.

0       _    _

-  (._  _% 00 (.  o  0.
o - _ (.  (.4  o.  0 0 0 0   0 o

I-I

00
(.4

Co 1-1  it  _   _N

-o 4 _ .4 _O 4 - o -4 -

o n- - D0o

2        0E

oco

_<   2       -

< < D m O X< a <a

0

'a
0

E

1)

12
0
0
C)

0
C

0

0

.0

2

CC

0
2

0

C)

Z.

E

.0

x

E

0

0

0

0 0

D0

.=a0

C)Y

.0
70
+ .O

su 0

I Q
1)

._

0+

1)

0

C)

0

Go
co

._

0

0

a

0

?

2_

0
C)

0

En

CO

0

2

1)

co
0

a

1)

0

(-

ca.

0
U

0

0
G

s

0
0

0.

= r

0

as0
0 0

= 0

= D.

0.

1

0

-1

0
'I

+

0-

03

+

+3
0Z

+

-  _  o - > (4  000 -W   v

(-.

C-

0%

(.4

- 0   . C   CD  CDC1
en 0   0 0D 0 O  _

*-_}o - -O rt o~ O -% O -cO -

a, 0 - e    - 0   -, C
1- I-, -  C) 0    0 0 -  o-,

1-       -    -   "I m -

'.r- o v- 4n  00  CD0CD0C

en

IC  CI  en  X 0

-  -  --o   0 o  - 0-

C-  D o F- -   -  -

F - 0 - 0,  _4 _
00 ~ ~ ~ ~

ID  - c  -  v o t-Z, -   0   C, -  0

00
C'A~~~~~~~~~~~~~.

00
(.4

fs o  It CD o )  CD  CD o 1 aoo

- -   - o  0 0 _-   (.-

t- CIDN 0   00 00 C

en -  -- d2  O2  O  Wm  en

on  - F  r-  -, (   -0 C   C  0 C

C'4 ~ ~  0-
00  00  (.  (4  0

o~~~~~ ~~~~   WU   OM own _

___-< __o  -  -  o -
s -   (.4  (   0  1- 0 0   - 0 0 0 0_

s ~>      _)   F

_?_U U Z> _  _O   o  _  _

(-4

o.4

10%

(.4

0

-
0

k ;

+l

tk
Q..

I+

I
II

-

0m

+

._
0

x
0

E

0
-o

._

0

0

ea

D

._

?

2

._
0
u

0

._

CO

0

1)

C)

a

1)

as

a._

00

U

0

0

a

0

0

0

.0
1-

E

2

*0

0
1)

E

C)
C)

-o

0

0

C4
0

2

& .

0

._
C-

0

E

._

_._
0-

._ .0

3 .D

C)>D

.2 X
C) - o

A E

0e

+- o0

So 0

O0'-

2+
0

0+'

0.0

= c

co

3)

0 0

1)0

0 C
=

IN VITRO RESISTANCE-MODIFYING ACTlVITY OF CYCLOSPORINS  15

Table IV Relationship between resistance modifier response rates and the percentage of samples
showing synergistic interactions, for cyclosporin A (CsA) or SDZ PSC 833 (PSC) in combination

with doxorubicin (Dox) or vincristine (Vcr)

Correlation
Responders      Synergy        O/E vs

(%)a           (%}b       change in SI'    n       P-value

Dox 0.5 Lg ml- '

+ CsA I jg ml-'         28             33           -0.75        296      <0.001
+CsA 3 tgml-'           49             41           -0.51        132      <0.001
+ PSC 1 Lg ml'          20             26           -0.77        269      < 0.001
+PSC 3iLgml'            50             47           -0.75        129      <0.001
Vcr 0.1 jg ml-'

+ CsA I Lg ml'          33             38           -0.67        229      < 0.001
+CsA 3pggml'            53             41           -0.54         81      <0.001
+PSC lpgml-'            20             29           -0.82        225      <0.001
+PSC 3 igml-'           49             40           -0.82         78      <0.001

'Percentage of samples showing >25% decrease in cytotoxic drug-induced survival index (SI)
in presence of modifier, Range of s.d. = 2-6%. bPercentage of samples with observed/expected
ratio (O/E) <0.8 (see Materials and methods). Range of s.d. = 3-5%. cChange in SI refers to
the percentage change in cytotoxic drug-induced SI caused by the cyclosporin.

Table V Relationship between resistance modifier response rates for cyclosporin A (CsA) and SDZ

PSC 833 (PSC) in combination with doxorubicin (Dox), and Dox sensitivity

LDR?                    IDR                   EDR

Dox 0.5 iLgml-'    +    ++   Total   n     +    ++    Total  n    +    ++    Total   n
+ CsA I gg ml-'    25   11    36    174    18     8    26    85    7    3    10,    60
+CsA 3.tgml-'      42   24    66     67    33    15    48    39   11    6    17b    36
+PSC Iigml-'       20    5    25    154     7     4    jlb  68     9    5    14     55
+PSC 3pgml-'       40   28    68     65    28    21    49    39   10    3    13b    31

The table shows the percentage of samples showing 25-50% (+) or > 50% (+ +) decrease in
Dox-induced survival index (SI) in the presence of modifier. The total response rate (+ and + +) is
indicated in bold numbers. 'LDR (low drug resistance) indicates samples showing SI values< median,
IDR (intermediate drug resistance) those showing SI values between median and median + 1 s.d. and
EDR (extreme drug resistance) those showing SI values> median + I s.d. The median and s.d. were
calculated from the Dox responses in all samples. "The response rates in the EDR group were
significantly lower than in both the LDR group and the IDR group for I and 3 gLg ml1 - CSA and for
3 jig ml- ' PSC. The difference between the LDR and IDR group was significant for 1 jug ml-' PSC.

0

0-

-

0

co

0

0.
0

0
0-

0

D

0

0.

o
0

CD

Vcr+L;sA 1 Vcr+LSA X Vcr+rbU I Vcr+rbi- J

Drug combination

Frgwe 2 Response rates to CsA and PSC in combination with
Dox or Vcr for previously untreated (-) or treated (0) patients,
presented as mean values ? s.e.m. Statistically significant
differences are indicated.

RMs. A tendency to increasing response rates in the treated
category was observed, and the differences were statistically
significant for the combinations Dox + PSC 3 g ml-' and
Vcr + I or 3 ig ml -' CsA or I pg ml-' PSC (P <0.05).

Relationship to cytotoxic drug sensitivity

In Table V the samples were divided into LDR, IDR and
EDR groups with mgard to Dox sensitivity. The highest RM
response rates were found in the LDR category followed by
IDR and EDR. In the EDR group significantly lower
resistance-modifying activity than in both the LDR group
(P<O.OOl) and the IDR group (P<0.05) was observed for
Dox + CsA   1 and 3 Lg ml ' and Dox + PSC   3 Lg mlP '.
Between the LDR and IDR groups statistical difference was
observed for Dox + PSC I ug mli (P <0.05).

In the present study the RM effects were measured at fixed
concentrations of the chemotherapeutic drugs instead of
measuring changes in the concentration required to achieve a
fixed effect (i.e. IC5), a procedure often used in experiments
on drug-resistant cell lines. One reason was that from many
of the samples not enough tumour cells were obtained to
permit detailed dose-response analysis. Measuring variable
effects at a fixed dose in highly heterogeneous patient popula-
tions also resembles the clinical phase II setting. The concent-
rations used were carefully chosen to produce maximal scat-
ter of SI values in a large panel of samples in order to have
the highest probability to distinguish sensitive from resistant
samples (Larsson et al., 1992a). Furthermore, the individual
intra-assay variation (CV) was used to statistically define the

r

7

16    H. FRIDBORG et al.

response criteria. Since the primary objective was to investi-
gate the relative subpanel specificity of RM effects under
identical in vitro conditions, we find the present approach
adequate for that purpose.

At I ytg mn' CsA and PSC, the cyclosporins alone had
little effect, whereas at 3 iLg ml1 ' a small cytotoxic effect was
noted in the haematological tumours. For CsA this was most
apparent in the CLL/NHL group, which is in accordance
with a previous study (Larsson et al., 1992h). In the majority
of cases the effect of the cyclosporins alone could not explain
the effect observed for the combinations since there was a
close correspondence between the percentage decrease in
cytotoxic drug-induced SI after addition of RM and the
calculated observed/expected ratios according to the multipli-
cative concept for drug interactions. The proportion of sam-
ples with synergistic interactions also closely paralleled the
response rate to addition of RM. However, 3 jg ml-' CsA in
combination with Dox or Vcr showed a slightly weaker
correlation between the RM-mediated percentage decrease in
cytotoxic drug-induced SI and observed/expected ratios com-
pared with the other RM concentrations tested. This may be
due to some effect of CsA alone, and indicates that, for at
least some tumours and at higher concentrations, the effect of
CsA alone may contribute to the cytotoxic efficacy of a CsA
and cytotoxic drug combination.

The present results demonstrate that CsA and PSC show
high   in  vitro  resistance-modifying  activity  against
haematological tumours, in which Pgp has been implicated in
drug resistance (Hall & Cattan, 1991; Nooter & Herweijer,
1991). The solid tumours were mostly considerably less re-
sponsive. However, both paediatric solid tumours and
ovarian carcinoma showed substantial response to both CsA
and PSC at the lower concentration. Included in the paediat-
ric solid group were four sarcomas and one neuroblastoma,
which are tumour types in which Pgp has been shown to be
liiked to the outcome of therapy (Chan et al., 1990, 1991).
However, this observation should be interpreted with caution
because of the limited number of samples tested so far.

Not all diagnoses showed responses in correspondence
with known patterns of Pgp expression. Renal and adreno-
cortical carcinoma showed no responses at all despite
reported high intrinsic expression of Pgp (Goldstein et al.,
1989). Also, adult sarcomas and breast carcinoma have been
reported to express intermediate levels of Pgp (Nooter &
Herweijer, 1991), but showed little response to RM addition
in the present study. In contrast, ALL, dominated by
paediatric samples, showed high response rates to CsA and
PSC but has been reported to express the Pgp in only a small
fraction of cases (Pieters et al., 1992). However, the role of
Pgp in childhood ALL still appears controversial (Gosauguen
et al., 1993).

Clinical documentation on the RM efficacy of cyclosporins
is sparse. In a study of CsA + vinblastine given to 22 patients
with renal cell carcinoma, no responses were observed
(Rodenburg et al., 1991). In a trial of 23 patients with
metastatic colon carcinoma only 1 out of 23 responded with
a partial remission to an epirubicin + CsA combination
(Murren et al., 1991). In AML, on the other hand, good
responses were reported for patients resistant and refractory
to AraC + daunorubicin after addition of CsA (List et al.,
1993). Promising tumour responses were also obtained in
myeloma patients using VAD    (Vcr+ Dox+prednisolone)
+ CsA (Sonneveld et al., 1992). Furthermore, in a recent
literature review of clinical investigations of MDR-reversing
agents, presenting results from more than 350 treated
patients, many leukaemias, lymphomas and myelomas were
considered to be potentially responsive whereas, in solid

tumour types, only a few patients appeared to benefit from
the RM treatment (Raderer & Scheithauer, 1993). Although
these observations are in accordance with the present in vitro
results, the true value of the present in vitro predictions will
have to await the completion of phase II trials representing a
broader array of diagnoses with careful monitoring of

systemic exposure of both the cytotoxic drugs and the
RMs.

Interestingly, we found no increased potency of PSC com-
pared with CsA, despite previous reports of a 10- to 20-fold
difference in favour of PSC observed in some (Gaveriaux et
al., 1991; Jonsson et al., 1992), but not all (Friche et al.,
1992), MDR cell lines. There is no difference in storage
stability of the stock solutions or in stability under the
FMCA culture conditions between the cyclosporins (not
shown). Moreover, Pgp-expressing MDR cell lines, analysed
regularly at our laboratory under identical conditions, are
more susceptible to PSC than to CsA (Jonsson et al., 1992).
These facts, together with the high resistance-modifying
activity noted in ALL, indicate the presence of additional
cyclosporin-sensitive mechanisms of resistance other than
Pgp in the clinical specimens. The non-Pgp-mediated MDR
phenotype has been shown to be sensitive, at least partly, to
RMs (Baas et al., 1990; van Kalken et al., 1991), and alterna-
tive target proteins have recently been identified (Slovak et
al., 1993). The fact that some of the tumour types originating
from Pgp-expressing tissues, and which are known to express
high Pgp levels, do not show any response to PSC and CsA
clearly indicates that RM-insensitive mechanisms of resis-
tance are also important, at least for these types of tumours.
In this context, one should note that clinically achievable
steady-state concentrations of CsA correspond to the in vitro
concentrations tested (1-3 1g ml-') (Sonneveld et al., 1992).
The Dox and Vcr concentrations used (EDCCs) correspond
roughly to the clinically achievable peak plasma concentra-
tion (Alberts & Chen, 1980). Using bioassay techniques
under the FMCA conditions these concentrations were
shown to give an in vitro exposure > 5-fold higher than what
is clinically achievable (to be published). Consequently, the
lack of response in some of the solid tumour types does not
seem to be related to insufficient exposure to the RM or the
cytotoxic drug.

Although a tendency to an increased resistance-modifying
activity was observed in samples from previously treated
patients, the response rate of those from untreated patients
was also substantial. Interestingly, the highest response rates
to RMs were found in the most drug-sensitive group of
samples, the LDR group, whereas the EDR group showed
significantly lower response rates. The low frequency of
responders to RMs in the EDR population thus further
underlines the virulent nature of this in vitro-defined type of
resistance (Weisenthal et al., 1991). These observations sug-
gest that RM treatment may be especially advantageous as
adjunct to up-front therapy in tumour types showing some
degree of cytotoxic drug sensitivity.

In summary, the present results indicate differential in vitro
activity of cyclosporins among the tumour types tested with
regard to the potentiating effect on the activity of two cyto-
toxic drugs. If this tumour type-specific activity is substan-
tiated.in clinical trials, non-clonogenic assays like the FMCA
may be useful in evaluation of new RMs and in targeting
specific diagnoses and patients for initial clinical trials of
RMs.

This study was supported by grants from the Swedish Cancer Society
(2695-B93-06XCC), The Children Cancer Foundation of Sweden and
The Lions Cancer Foundation. The skilful technical assistance of
Charlotta Sandberg and the cytopathological expertise provided by
Manuel de la Torre are gratefully acknowledged.

Abbrevtioin: MDR, multidrug resistance; Pgp, P-glycoprotein; RM,

resistance modifier, CsA, cyclosporin A; PSC, SDZ PSC 833; AML,
acute myelocytic leukaemia  ALL, acute lympocytic leukaemia;
CML, chronic myelocytic leukaemia; CLL, chronic lymphocytic
leukaemia; NHL, non-Hodgkin's lymphoma; LDR, low drug resist-
ance; IDR, intermediate drug resistance; EDR, extreme drug resist-
ance; CV, coefficient of variation; s.d., standard deviation; s.e.m.,
standard error of the mean.

IN VITRO RESISTANCE-MODIFYING ACTIVITY OF CYCLOSPORINS  17

Referems

ALBERTS. D. & CHEN. G. (1980). Tabular summary of pharmaco-

kinetic parameters relevant to in vitro assay. In Cloning of Human
Tumor Stem Cells, Salmon, S. (ed.) pp. 351-359. Alan R. Liss:
New York.

BAAS. F.. JONGSMA. A.. BROXTERMAN. H.. ARCECI, R., HOUSMAN,

D., SCHEFFER, G., RIETHORST, A.. vAN GROENIGEN, M., vAN
NIEUWINT. A. & JOENJE. H. (1990). Non-P-glycoprotein
mediated mechanism for multidrug resistance precedes P-glyco-
protein expression during in vitro selection for doxorubicin resist-
ance in a human lung cancer cell line. Cancer Res., 50,
5392-5398.

BORST. P. (1991). Genetic mechanisms of drug resistance. Rev.

Oncol., 4, 87-105.

BOSANQUET. A. (1991). Correlations between therapeutic response

of leukemias and in vitro drug sensitivity assay. Lancet, i,
711-714.

CHAN. H.. HADDAD. G.. THORNER. G., DEBOER, G., LIN, Y., OND-

RUSEK. N.. YEGER, H. & LING. V. (1991). P-glycoprotein expres-
sion as a predictor of the outcome of therapy in neuroblastoma.
N. Engl. J. Med., 325, 1608-1614.

CHAN. H.. THORNER. P.G.H. & LING. V. (1990). Immunohisto-

chemical detection of P-glycoprotein: prognostic correlation in
soft tissue sarcoma of childhood. J. Clin. Oncol., 8, 689-704.

COLTON. T.C. (1974). Statistics in Medicine. Little, Brown: Bos-

ton.

DALTON. W.S.. GROGAN. T.M., MELTZER. P.S.. SCHEPER, RJ..

DURIE. B.G.M.. TAYLOR. C.W.. MILLER. T.P. & SALMON, S.E.
(1989). Drug-resistance in multiple myeloma and non-Hodgkin's
lymphoma: detection of P-glycoprotein and potential circumven-
tion by addition of verapamil to chemotherapy. J. Clin. Oncol., 7,
415-424.

FORD. J. & HAIT. W. (1990). Pharmacology of drugs that alter

multidrug resistance in cancer. Pharmaol. Rev., 42, 156-199.

FRICHE. E. JENSEN. P. & NISSEN. N. (1992). Comparison of cyclo-

sporin A and SDZ PSC 833 as multidrug-resistance modulators
in  daunorubicin-resistant  Ehrlich  ascites  tumor.  Cancer
Chemother. Pharmacol., 30, 235-237.

GAVERIAUX. C.. BOESCH. D., JACHEZ. B.. POLLINGER, P., PAYNE,

T. & LOOR. F. (1991). SDZ PSC 833, a non-immunosuppressive
cyclosporin analog, is a very potent multidrug-resistance
modifier. J. Cell. Pharmacol., 2, 225-234.

GOLDSTEIN, LJ. GALSKI, H., FOO, A., WILLINGHAM, M., LAI, S.,

GAZDAR, A.. PIRKER, R.. GREEN, A.. CRIST, W., BRODEUR,
G.M.. LIEBER, M., COSSMAN, J., GOTTESMAN, M.M. & PASTAN,
I. (1989). Expression of a multidrug resistance gene in human
cancers. J. Nati Cancer Inst., 81, 116-124.

GOSAGUEN. J.. DOSSOT, J.. FARDEL, O., LE MEE. F., LE GALL, E.,

LEBLAY. R., LEPRISE. P.. CHAPERON. J. & FAUCHET, R. (1993).
Expression of the multidrug resistance-associated P-glycoprotein
(P-170) in 59 cases of de novo acute lymphoblastic leukemia:
prognostic implications. Blood, 81, 2394-2398.

HALL A. & CATTAN. A. (1991). Drug resistance mechanisns in

leukemia. Bailiere's Clin. Hematol., 4, 655-677.

JONSSON, B., NILSSON, K, NYGREN, P. & LARSSON, R_ (1992). SDZ

PSC-833 - A novel potent in vitro chemosensitizer in multiple
myeloma. Anti-Cancer Drugs, 3, 641-646.

LARSSON, R., KRISTENSEN, J., SANDBERG, C. & NYGREN, P.

(1992a). Laboratory determination of chemotherapeutic drug
resistance in tumour cells from patients with leukemia using a
fluorometric mkroculture cytotoxicity assay (FMCA). Int. J.
Cancer, 50, 177-185.

[ARSSON, R. CSOKA, K., KRISTENSEN, J., NLLSSON, K. & NYGREN,

P. (1992b). Selective cytotoxic activity of cyclosporins against
tumor cells from patients with chronic lymphocytic leukemia.
Eur. J. Pharmacol., 53, 23-30.

LARSSON, R. & NYGREN, P. (1993). Prediction of individual patient

response to chemotherapy using drug specific cut-off lmits and a
Bayesian model. Anticancer Res., 13, 1825-1830.

LARSSON, R., FRIDBORG, H., CSOKA, K, LILIEMARK, J., KRISTEN-

SEN, J., DE LA TORRE, M. & NYGREN, P. (1994). In vitro activity
of 2-chlorodeoxyadenoside (CdA) in primary cultures of
hematological and solid tumors. Eur. J. Cancer. (in press).

LIST, A., SPIER, C., GREER, J., WOLFF, S., HUTTER, J., DORR, R.,

SALMON, S., FUTSCHER, B., BAIER, M. & DALTON, W. (1993).
Phase I/II trial of cyclosporine as a chemotherapy-resistance
modifier in acute leukemia. J. Clin. Oncol., 11, 1652-60.

MILLER, T., GROGAN, T., DALTON, W., SPIER, C., SCHEPER, R. &

SALMON, S. (1991). P-glycoprotein expression in malignant lym-
phoma and reversal of clinical drug resistance with chemotherapy
plus high-dose verapamil. J. Clin. Oncol., 9, 17-24.

MURREN, J., GANPULE, S., SARRIS, A., DURIVAGE, H., DAVIS, C.,

MAKUCH, R & HANDSCHUMACHER, R_ (1991). A phase II trial
of cyclosporin A in the treatment of refractory metastatic colo-
rectal cancer. Am. J. Clin. Oncol., 14, 208-210.

NOOTER, K. & HERWEUJER, H. (1991). Multidrug resistance (mdr)

genes m human cancer. Br. J. Cancer, 63, 663-669.

NYGREN, P., FRIDBORG, H., CSOKA, K., SUNDSTR5M, C., DE LA

TORRE, M., KIRSTENSEN, J., BERGH, J., HAGBERG, H.,
GLIMELIUS, B., RASTAD, J., THOLANDER, B. & LARSSON, R.
(1994). Detection of tumour-specific cytotoxic drug activity in
vitro using the fluorometric microculture cytotoxicity assay and
primary cultures of tumour cells from patients. Int. J. Cancer, 56,
715-720.

NYGREN, P. & LARSSON, RI (1991). Differential in vitro sensitivity of

human tumor and normal cells to chemotherapeutic agents and
resistance modulators. Int. J. Cancer, 48, 598-604.

PENNOCK, G., DALTON, W., ROESKE, W., APPLETON, C., MOSLEY,

K., PLEZIA, P., MILLER, T. & SALMON, S. (1991). Systemic toxic
effects associated with high-dose verapamil infusion and
chemotherapy administration. J. Natl Cancer Inst., 83,
105-110.

PIETERS, R, HONGO, T., LOONEN, A., HUISMANS, D., BROXTER-

MAN, H., HAHLEN, K. & VEERMAN, A. (1992). Different types of
non-P-glycoprotein mediated multiple drug resistance in children
with relapsed acute lymphoblastic klukemia. Br. J. Cancer, 65,
691-697.

RADERER, M. & SCHEITHAUER, W. (1993). Clinical trials of agents

that reverse multidrug resistance. Cancer, 72, 3553-3563.

RODENBURG, C., NOOTER, K., HERWEIYER, H., SEYNAEVE, C.,

OOSTEROM, R, STOTER, G. & VERWEUI, J. (1991). Phase 2 study
combining vinblastine with cyclosporin A to circumvent resist-
ance in renal cell cancer. Ann. Oncol., 2, 305-306.

SLOVAK, M., HO, J., BHARDWAJ, G., KURZ, E, DEELAY, R_ & COLE,

S. (1993). Locahsation of a novel multidrug resistance-associated
gene in the HT 1080/DR4 and H69AR human tumor cell lines.
Cancer Res., 53, 3221-3225.

SONNEVELD, P., DURIE, B., LOKHORST, H., MARIE, J., SOLBU, G.,

SUCIU, S., ZITITOUN, R., LOWENBERG, B. & NOOTER, K_ (1992).
Modulation of multidrug resistant multiple myeloma by cyclo-
sporin. Lancet, 346, 255-259.

TWENTYMAN, P. (1988). A possible role for cyclosporins in cancer

chemotherapy. Anticancer Res., 8, 985-994.

VALERIOTE, F. & LIN, H. (1975). Synergistic interaction of anti-

cancer agents: A cellular perspective. Cancer Chemother. Rep., 59,
895-900.

VAN KALKEN, C., PINEDO, H. & GIACCONE, G. (1991). Multidrug

resistance from a clinical point of view. Eur. J. Cancer, 27,
1481-1486.

WEISENTHAL, L. (1991). Predictive assays for drug and radiation

resistance. In Hwnan Cancer in Primary Culture, a Handook,
Masters, J. (ed.) pp. 103-147. Kluwer Academic Publishers: Dor-
drecht, The Netherlands.

WEISENTHAL, L., DILL, P. & BIRKHOFER, M. (1991). Accurate

identification of disease-specific activity of antineoplastic agents
with an in vitro fresh tumor assay measuring killing of largely
non-dividing cells. Proc. Am. Ass. Cancer Res., 32, 384.

YING TAN, Y., EPSTEIN, L. & ARMSTRONG, R. (1988). In vitro

evaluation of 6-thioguanine and a-interferon as a therapeutic
combination in HL-60 and natural killer cells. Cancer Res., 43,
258-264.

				


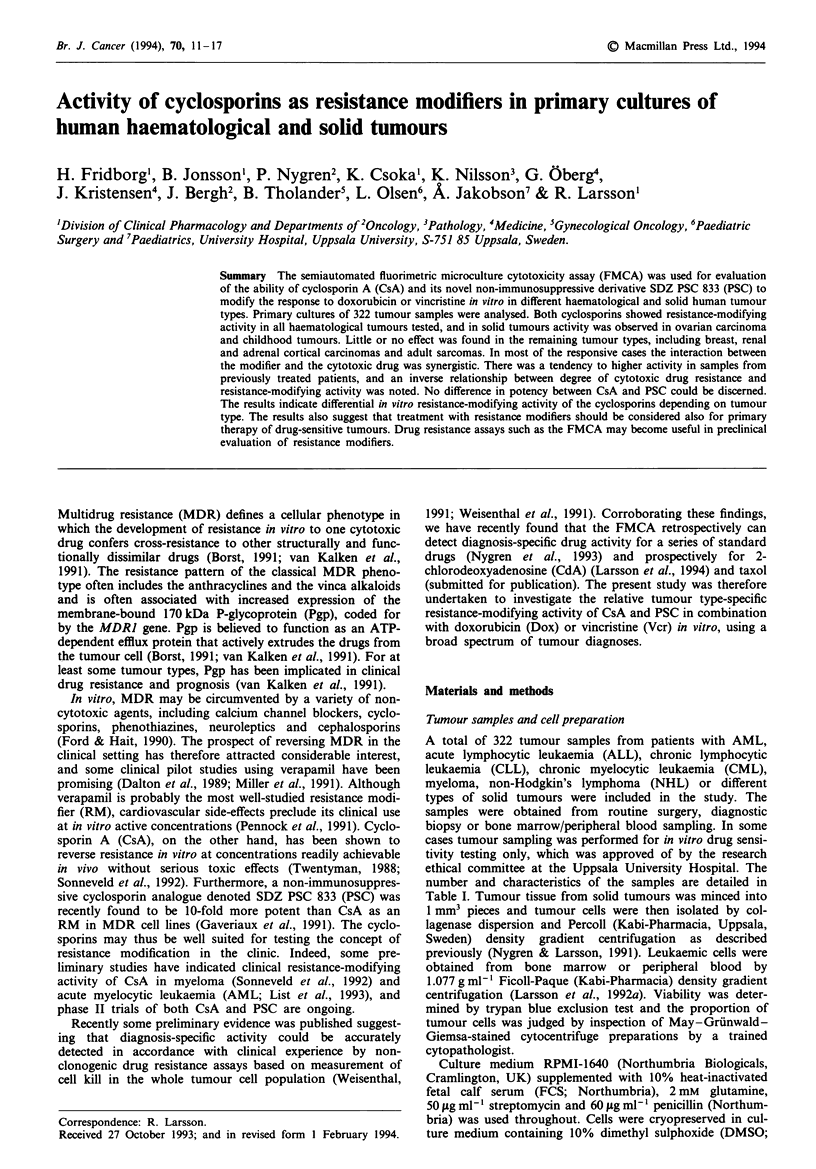

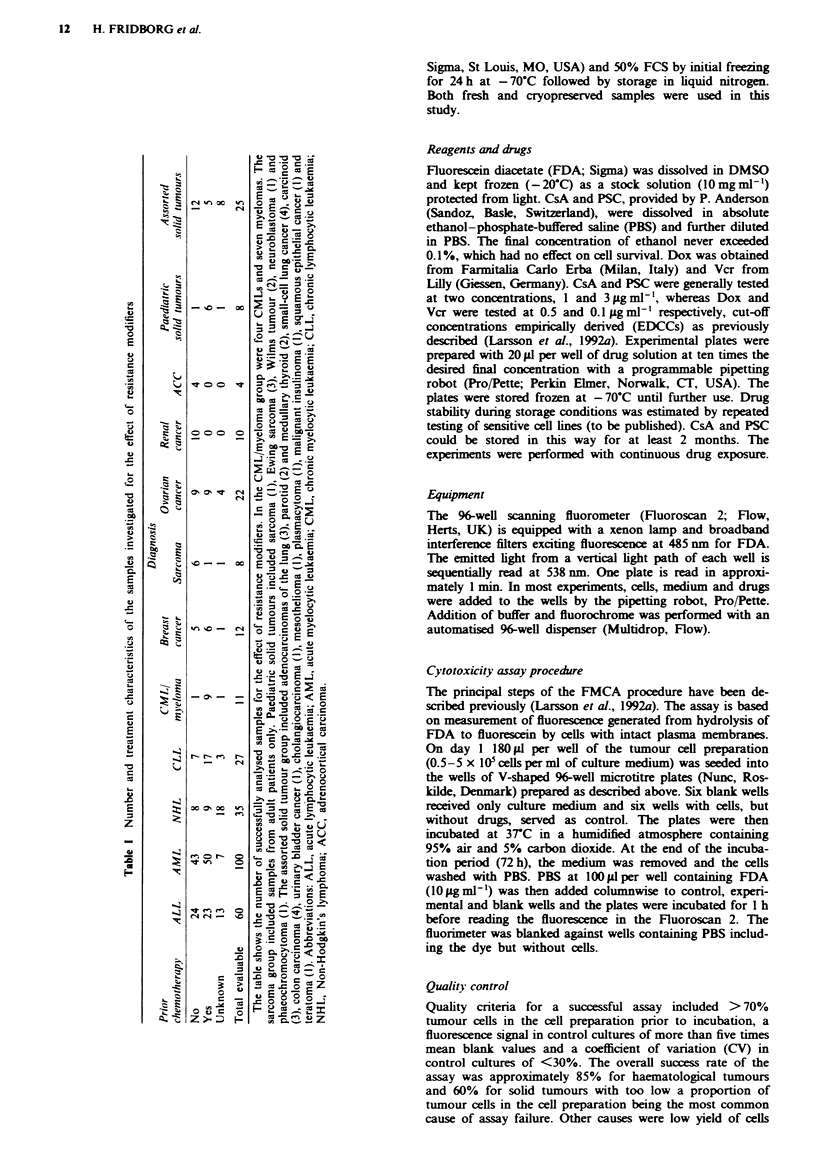

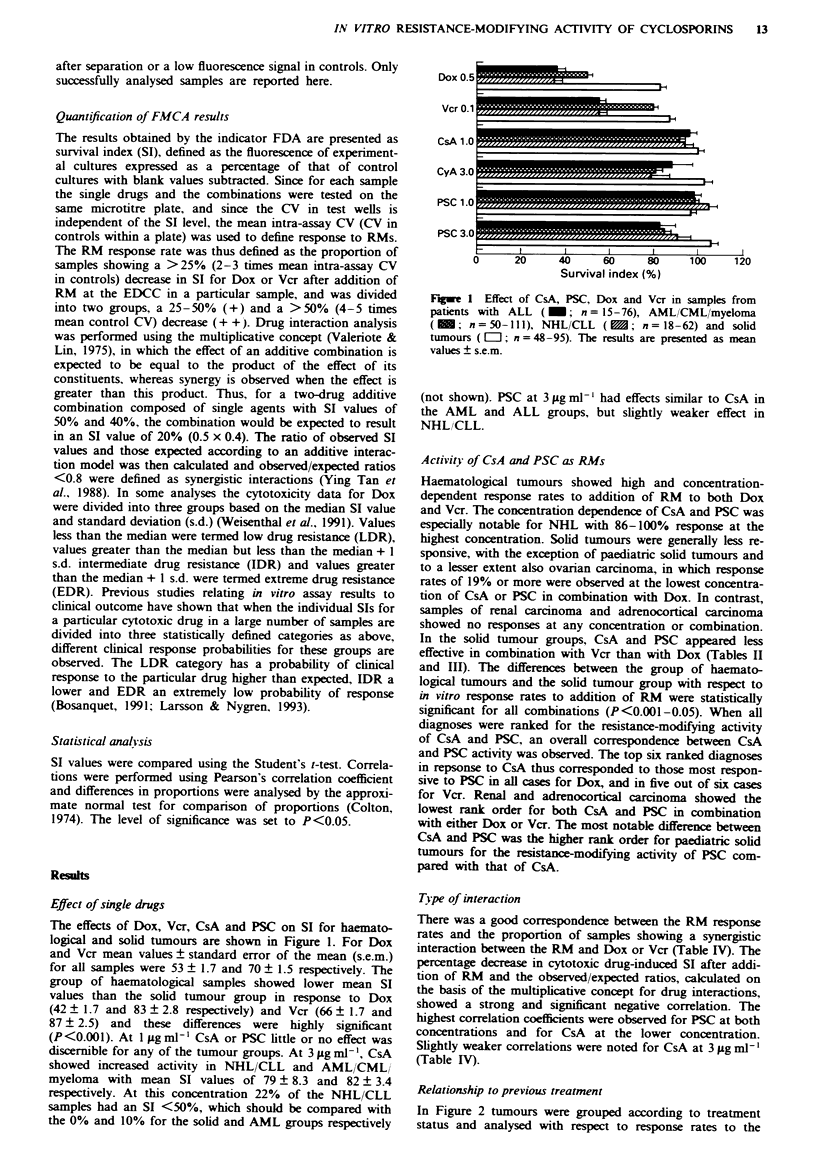

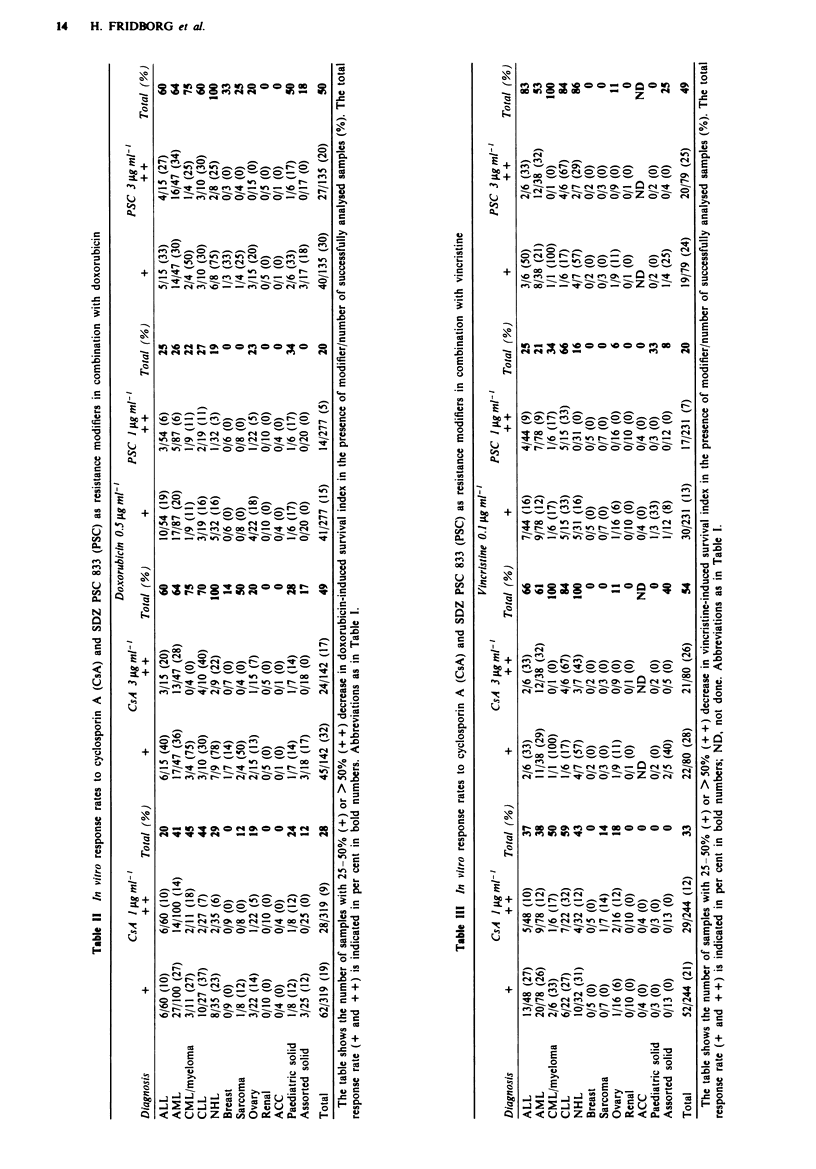

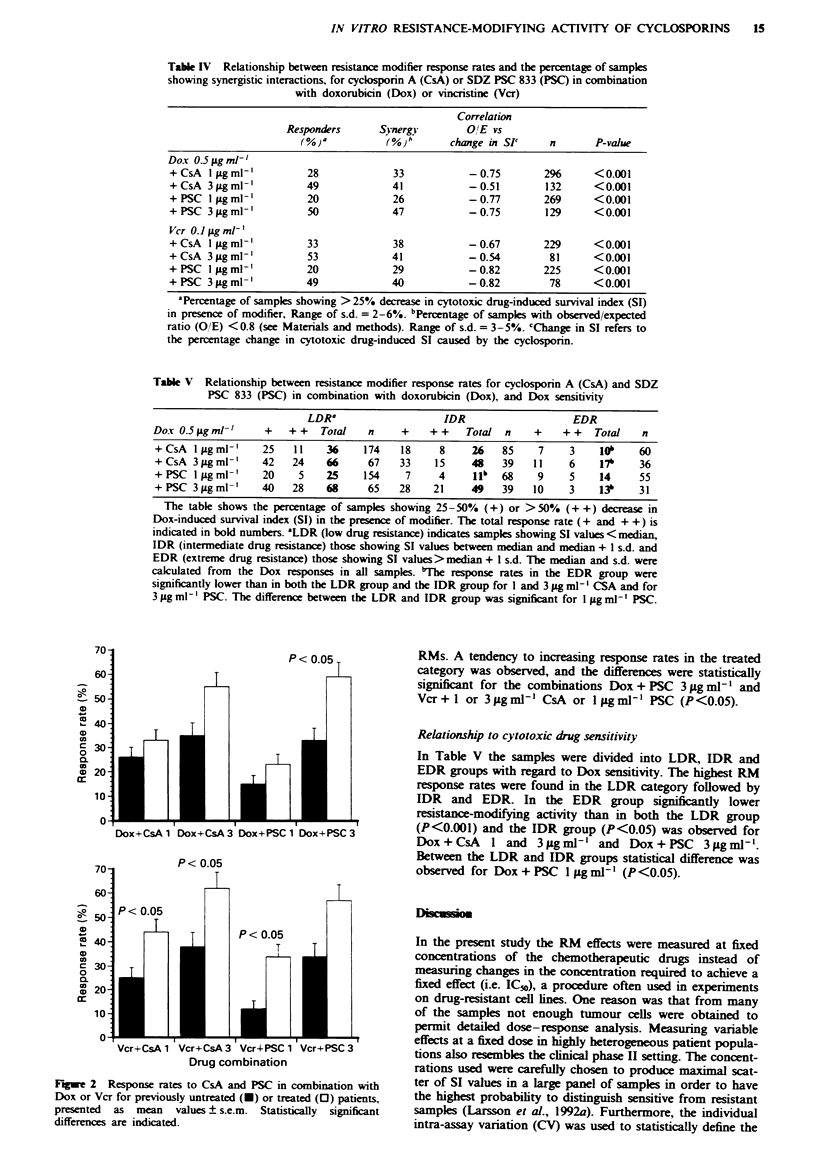

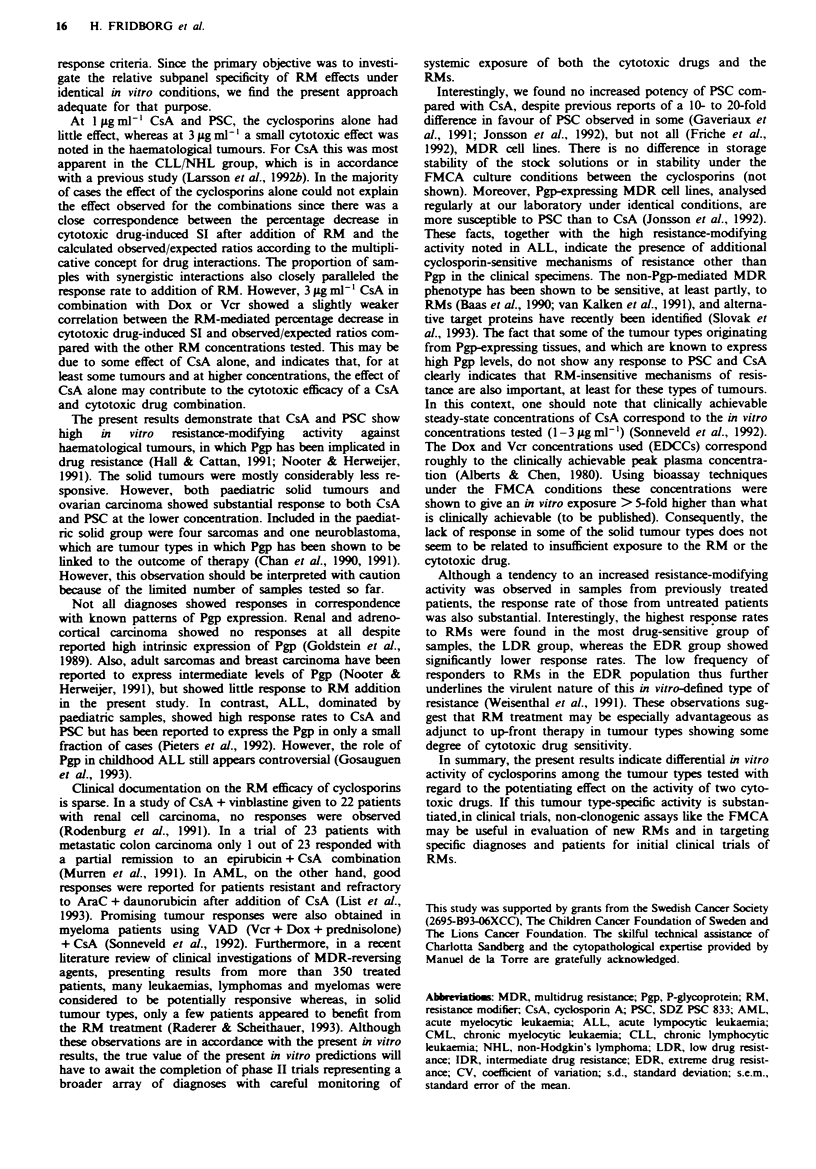

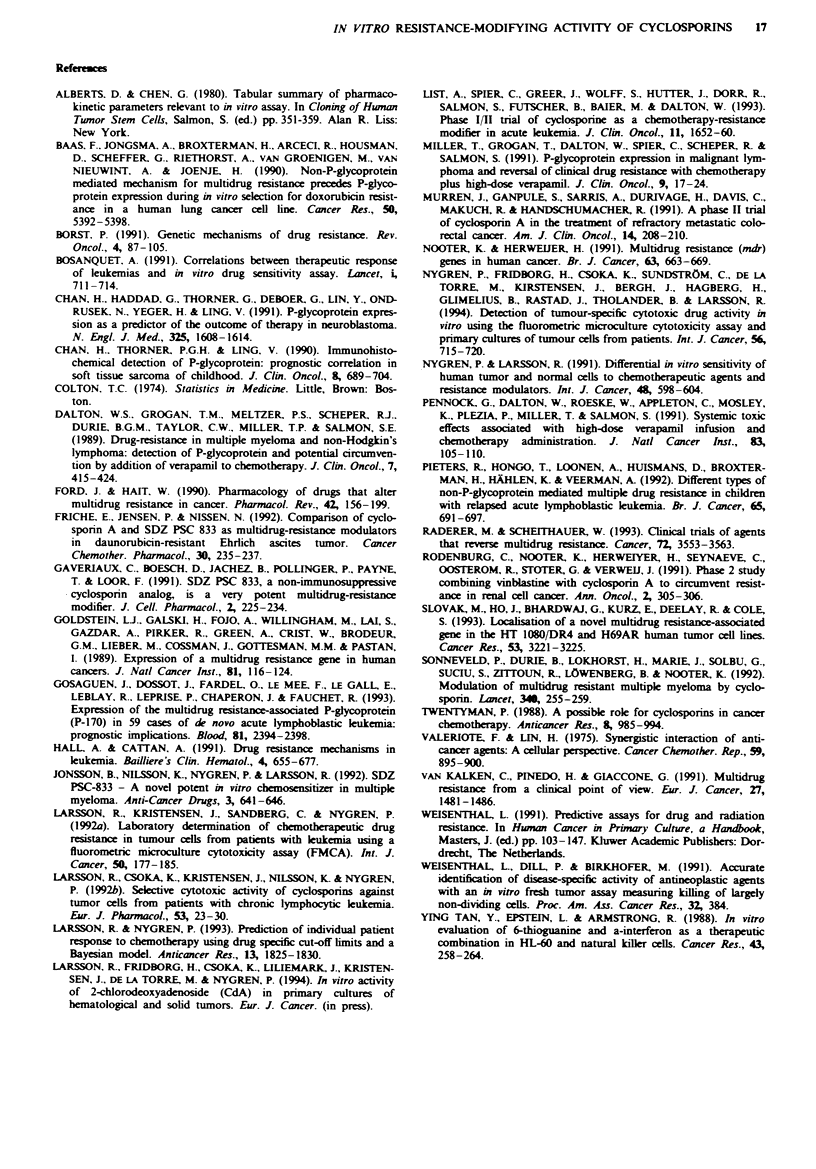

